# Association of Breastfeeding and the Federal Poverty Level: National Survey of Family Growth, 2011–2013

**DOI:** 10.1155/2016/9783704

**Published:** 2016

**Authors:** R. Constance Wiener, Usha Sambamoorthi, Sarah E. Hayes, Ilana R. Azulay Chertok

**Affiliations:** 1 Department of Dental Practice and Rural Health, School of Dentistry, West Virginia University, 104A Health Sciences Center Addition, P.O. Box 9448, Morgantown, WV 26506, USA; 2 Department of Pharmaceutical Systems and Policy, West Virginia University School of Pharmacy, Robert C. Byrd Health Sciences Center (North), P.O. Box 9510, Morgantown, WV 26506-9510, USA; 3 West Virginia University Department of Psychology, 53 Campus Drive, P.O. Box 6040, Morgantown, WV 26506, USA; 4 University of North Carolina Charlotte School of Nursing, CHHS-420, Charlotte, NC 28223, USA

## Abstract

Breastfeeding is strongly endorsed in the Healthy People 2020 goals; however, there remain many disparities in breastfeeding prevalence. The purpose of this study was to examine the association between breastfeeding and the Federal Poverty Level in the United States. Data from 5,397 women in the National Survey of Family Growth 2011–2013 survey were included in this study. The data were analyzed for descriptive features and logistic regressions of the Federal Poverty Level on breastfeeding. There were 64.1% of women who reported breastfeeding. Over one-third (35.2%) of women reported having a household income of 0–99% of the Federal Poverty Level. There were 15.2% of women who reported an income of 400% and above the Federal Poverty Level. With statistical adjustment for maternal age, race/ethnicity, education, marital status, parity, preterm birth, birth weight, insurance, and dwelling, the Federal Poverty Level was not significantly associated with breastfeeding. In this recent survey of mothers, Federal Poverty Level was not shown to be a significant factor in breastfeeding.

## 1. Introduction

The American Academy of Pediatrics recommends the exclusive breastfeeding of infants to age six months, with continued breastfeeding (complemented by solid foods) for one year or longer [1]. The United States (US) Department of Health and Human Services recognizes the public health benefits of breastfeeding and has nine breastfeeding-related objectives for Healthy People 2020 goals [2]. These objectives include increasing the number of infants having ever been breastfed from the baseline of 74.0% to 81.9%; increasing the number of infants who are breastfed to age 6 months from the baseline of 43.5% to 60.6%; and increasing the number of facilities that provide recommended care for lactating mothers and newborns from a baseline of 2.9% to 8.1% [2].

There are many barriers to breastfeeding that have been reported in earlier studies including lack of support [3, 4], public beliefs [3], difficulty with the breast pump [5], young age of mother, less education, unmarried status, fear of embarrassment, fear of being fired, privacy, sexualization of the breast, change in appearance of the breast, pain, bleeding, difficulty latching-on, insufficient milk, race/ethnicity, and low income [6]. In a population-based study examining the influence of poverty and participation in the federal Special Supplemental Nutrition Program for Women, Infants, and Children (WIC) in South Carolina, researchers found that WIC participation was the strongest predictor of lack of breastfeeding initiation in that state [7].

Women who participated in WIC programs faced additional barriers to breastfeeding [8]. One of the themes that emerged in a qualitative study of WIC counselors serving primarily African American families was that formula use was seen as a sign of wealth [9]. Prior to the recently revised WIC breastfeeding incentive program of augmented food packages for breastfeeding women, WIC participation had been associated with lower breastfeeding initiation and duration rates [10]. WIC credits can be used for supplemental formula, and many clients viewed the supplemental formula as more valuable than the offset of expanded food packages [8].

With goals in place and concerted efforts to increase breastfeeding rates, research results have been inconsistent regarding the association between family income and breastfeeding; some researchers indicate no association [11, 12], others support an association [13, 14], and others report equivocal results [15]. The aim of this study was to determine if there was an association between breastfeeding and the Federal Poverty Level (FPL) using data from the National Survey of Family Growth 2011–2013.

## 2. Methods and Materials

Data from the National Survey of Family Growth (NSFG) 2011–2013 data were used to conduct a cross-sectional secondary data analysis of the association of FPL and breastfeeding. The 2011–2013 survey is the NSFG’s 8th data file release since 1973 (National Survey of Family Growth 2015) [16]. The NSFG survey was specifically designed to determine family trends as well as differences among groups in family sizes, family structure, use of contraception, sexual activity, and infertility for use in designing health services and educational programs [16]. The sampling was a multistage probability-based national representative of US households [16]. Details of the survey are provided at the NSFG website, http://www.cdc.gov/nchs/nsfg/nsfg_2011_2013_puf.htm.

This study received the West Virginia University Institutional Review Board study acknowledgement (protocol number 1502572781). The research was conducted in accordance with prevailing ethical principles.

### 2.1. Study Population

Participants in the current study of the association between breastfeeding and FPL were women of childbearing age (14 to 44 years) who had completed NSFG 2011–2013 data for the following variables: breastfeeding; FPL status; and race/ethnicity. The sample size was 5,397 women.

### 2.2. Variable Definitions

The dependent variable was breastfeeding, defined as breastfeeding one week or more. (The definition did not include *intent* to breastfeed.) The variable was a dichotomized “yes” or “no” variable.

The independent variable was the FPL of the mother. FPL was provided by the NSFG as a recoded variable with five levels: 0–99% FPL, 100–199% FPL, 200–299% FPL, 300–399% FPL, and 400% and above FPL. Other sociodemographic and health variables included in the analyses were maternal age (14 to less than20 years; 20 to less than25 years; 25 to less than 30 years; or 30 to 44 years); maternal race/ethnicity (non-Hispanic white; non-Hispanic black; or Hispanic); maternal education (less than high school; high school graduate; some higher education; or Associate degree and above);marital status (married or single/divorced/separated/widowed); parity (first infant or 2 or more); preterm birth (yes, less than 37 weeks, or no, 37 weeks and above); low birth weight (yes, less than 2,500 grams; no, 2,500 grams and above); insurance (private or MediGap; Medicaid, CHIP, or a state-sponsored plan; Medicare, military, or other government plan; or single service, Indian Health Service, or not covered); and dwelling (urban, principal city; urban, other than principal city; or rural).

### 2.3. Analyses

Due to the complex sampling design of the NSFG 2011–2013, analyses were conducted to account for the computational units, strata, and final weights. SAS version 9.3^®^ (Cary, NC) software was used to determine the descriptive characteristics of the sample, and the relationship between breastfeeding and FPL in logistic regression. The model-based imputations in the NSFG 2011–2013 data for the variables chosen had a maximum imputation occurring with FPL (5.94%). An adjusted regression model was built which included maternal age, maternal race/ethnicity; maternal education; marital status; parity; preterm birth, birth weight; insurance; and dwelling.

## 3. Results

The study population was derived from the National Survey of Family Growth 2011–2013 data. There were 5,397 participants in this study. There were 3,302 (64.1%) participants who breastfed and 2,095 (35.9%) who did not breastfeed. There were 2,406 (35.3%) at 0–99% of the FPL; 1,324 (23.3%) at the 100–199% FPL; 701 (15.9%) at the 200–299% FPL; 414 (10.4%) at the 300–399% FPL; and 552 (15.1%) at or above the 400% FPL. The sample description is presented in Table 1.

In unadjusted logistic analysis, there were significant relationships between breastfeeding and income for all of the participants. In stratified analyses, there were significant associations between breastfeeding and income with maternal age in the 25–30 year category; maternal age in the 30 year and older category; maternal race/ethnicity; maternal education in the Associate degree and above category; parity in the second baby or above category; preterm birth; insurance in all categories except the Medicaid, Chip, State sponsored plan category; and urban dwelling. The results of the unadjusted logistic regressions are presented in Table 2.

The results of two logistic regression models on breastfeeding are presented in Table 3. The first model demonstrates the combined effect of entering maternal age, maternal education, marital status, and dwelling on the relationship between breastfeeding and FPL. FPL is attenuated and no longer significant in this parsimonious model. The complete model further attenuated the relationship.

There were no interactions with FPL and marital status, preterm birth, and dwelling when each variable was entered individually in the logistic regressions. There was a slight attenuation of the association of FPL and breastfeeding when maternal age and maternal education were entered individually in logistic regressions. It was the combined effects of the variables which altered the FPL-breastfeeding relationship. The results of the adjusted logistic regression analyses stratified for each category of maternal race/ethnicity, marital status, and preterm birth are presented in Table 4.

## 4. Discussion

The odds ratio for the association between FPL and breastfeeding failed to reach significance in an adjusted logistic regression with the covariates of maternal age, maternal race/ethnicity, maternal education, marital status, parity, preterm birth, low birth weight, insurance, and dwelling. Although studies exist which address only maternal low income and breastfeeding, there is a paucity of information concerning income disparity (concerning a more comprehensive and inclusive approach to income) and breastfeeding in the US. Nevertheless, similar results to support this study were found in a study of 10,519 women in California who gave birth between 1999 and 2001 in which Heck and colleagues [11] found that family income was not associated with breastfeeding. Lutter and Morrow [12] reported a trend in Africa, Asia, Latin America, and the Middle East over the previous two decades in which annual increases in breastfeeding were not associated with the gross national income of the participants’ respective countries.

Researchers conducting a study of three hospitals in Canada found conflicting results regarding breastfeeding initiation and maternal income. Overall breastfeeding initiation increased over time, although for one hospital the rate difference between maternal high income and low income decreased, for another hospital the rate difference remained the same, and for the third hospital the rate difference increased [15].

Supporting the association between breastfeeding rates and income, researchers using the 1999–2006 National Health and Nutrition Examination Surveys suggested that women with higher incomes were significantly more likely to breastfeed than women with low incomes [13]. In a large population-based ecological study in Ontario, Canada, women with higher incomes were more likely to breastfeed their infants than women with lower incomes [14].

Future studies are needed to further clarify the role of FPL on breastfeeding and to examine the role of supportive services, such as WIC, in encouraging breastfeeding. This study’s main limitation is that the data collected were self-reported. Self-reported measures are subject to social desirability bias, which occur when a participant responds in a way to appear more positive. Second, a causal interpretation cannot be applied as the cross-sectional design of the study does not indicate temporality. However, the study has several strengths. It is a large study using national data and the sampling used a multistage probability-based and representative design.

## 5. Conclusion

While FPL was significantly associated with lack of breastfeeding in unadjusted analyses, the role of FPL failed to reach significance in the adjusted regression analyses of the study, suggesting a need for all women to have breastfeeding initiation support. Women of childbearing age should be educated about the benefits of breastfeeding for themselves and their infants. The workers in supportive programs, such as WIC, are important in encouraging breastfeeding in their clients. Healthcare providers should continue to promote breastfeeding and to help meet Healthy People 2020 goals.

## Figures and Tables

**Table 1:** Descriptive statistics of participants in the National Survey of Family Growth (NSFG) 2011–2013.

	Yes breastfeeding		No breastfeeding		Total
	*N*	Wt Col%	*N*	Wt Col%	Total
All	**3,302**	**64.1**	**2,095**	**35.9**	**5,397**
Federal Poverty Level (FPL)					
0–99% of FPL	**1,270**	**18.9**	**1,136**	**16.3**	**2,406**
100–199% FPL	**811**	**14.7**	**513**	**8.6**	**1,324**
200–299% FPL	**487**	**10.9**	**214**	**5.0**	**701**
300–399% FPL	**299**	**7.4**	**115**	**3.0**	**414**
400% and above FPL	**435**	**12.2**	**117**	**3.0**	**552**
Maternal age (all)	3,302	64.1	2,095	35.9	5,397
14 to less than 20 years	472	6.6	468	7.2	940
20 to less than 25 years	1,065	18.1	827	13.2	1,892
25 to less than 30 years	995	21.2	504	10.0	1,499
30 years to 44 years	770	18.3	296	5.4	1,066
Maternal race/ethnicity (all)	3,302	64.1	2,095	35.9	5,397
Non-Hispanic white	1,535	39.7	802	19.3	2,337
Non-Hispanic black	561	7.1	752	8.6	1,313
Hispanic	1,206	17.3	541	7.9	1,747
Maternal education (all)	3,302	64.1	2,095	35.9	5,397
Less than high school	639	10.6	513	6.6	1,152
High school graduate	828	14.4	824	15.1	1,652
Some higher education	730	13.5	428	7.6	1,158
Associate degree and above	1,105	25.6	330	6.5	1,435
Marital status (all)	3,302	64.1	2,095	35.9	5,397
Single/separated/widowed/divorced	1,538	41.2	1,408	19.5	2,946
Married	1,764	22.9	687	16.3	2,451
Parity (all)	3,302	64.1	2,095	35.9	5,397
First infant	521	9.1	344	5.2	865
2 or more	2,781	55.0	1,751	30.7	4,532
Preterm birth (all)	3,254	64.1	2,068	35.9	5,322
Yes (less than 37 weeks)	390	6.8	315	5.5	705
No (37 weeks and above)	2,864	57.2	1,753	30.4	4,617
Low birth weight (all)	3,302	64.1	2,095	35.9	5,397
Yes (less than 2500 grams)	259	4.1	197	3.5	456
No (2500 grams and above)	3,043	60.1	1,898	32.3	4,941
Insurance (all)	3,302	64.1	2,095	35.9	5,397
Private or MediGap	1,486	35.8	626	14.7	2,112
Medicaid, CHIP, or a state-sponsored plan	779	9.8	821	11.0	1,600
Medicare, military, or other gv’t plans	219	3.4	109	1.8	328
Single service plan, IHS, or no coverage	818	15.2	539	8.4	1,357
Dwelling (all)	3,302	64.1	2,095	35.9	5,397
Urban, principal city	1,343	19.7	932	12.4	2,275
Urban, other than principal city	1,585	36.1	780	17.0	2,365
Rural	374	8.3	383	6.5	757

Note: based on 5,397 participants with infants from the National Survey of Family Growth 2011–2013. Preterm data were missing for 75 participants and the number and percentage of breastfeeding by these participants are not presented in the table.

*N*: number; CHIP: Children’s Health Insurance Program; gv’t: government; IHS: Indian Health Service; Wt Col%: weighted column percentage.

**Table 2:** Unadjusted odds ratios (OR) and 95% confidence intervals (CI) of Federal Poverty Level (FPL) categories (reference group = 0–99% FPL) from separate logistic regressions on breastfeeding National Survey of Family Growth (NSFG) 2011–2013.

	Number	100–199% FPL	200–299% FPL	300–399% FPL	400%+ FPL	Wald *p* value
All	**5,397**	**1.47 (1.09, 1.99)**	**1.89 (1.33, 2.70)**	**2.16 (1.37, 3.39)**	**3.51 (2.21, 5.59)**	**<0.001**
Maternal age						
14 to less than 20 years	940	1.25(0.72, 2.17)	1.64 (0.78, 3.44)	0.35 (0.11, 1.07)	0.82 (0.21, 3.21)	0.134
20 to less than 25 years	1,892	1.42 (0.95, 2.12)	1.58 (1.04, 2.40)	1.68 (1.02, 2.79)	1.67 (0.77, 3.60)	0.115
25 to less than 30 years	1,499	1.63 (0.99, 2.70)	2.29 (1.34, 3.94)	2.22 (1.20, 4.09)	2.91 (1.72, 4.92)	<0.001
30 to 44 years	1,066	1.32 (0.70, 2.48)	1.28 (0.63, 2.61)	2.48 (1.18, 5.22)	3.90 (1.96, 7.76)	0.001
Maternal race/ethnicity						
Non-Hispanic white	2,337	1.89 (1.18, 3.03)	2.57 (1.60, 4.12)	2.78 (1.62, 4.78)	4.18 (2.36, 7.40)	<0.001
Non-Hispanic black	1,313	1.63 (0.95, 2.81)	1.97 (0.93, 4.16)	4.83 (1.44, 16.21)	3.52 (1.25, 9.94)	0.005
Hispanic	1,747	1.16 (0.62, 2.01)	1.05 (0.40, 2.76)	0.78 (0.28, 2.17)	2.95 (1.27, 6.86)	0.023
Maternal education						
Less than high school	1,152	1.49 (0.90, 2.49)	1.36 (0.51, 3.68)	1.65 (0.23, 11.77)	0.23 (0.03, 1.83)	0.226
High school graduate	1,652	1.47 (0.86, 2.50)	1.73 (0.83, 3.62)	1.26 (0.46, 3.43)	1.59 (0.73, 3.46)	0.448
Some higher education	1,158	1.49 (0.86, 2.57)	1.28 (0.67, 2.45)	1.07 (0.37, 3.15)	2.07 (0.78, 5.51)	0.442
Associate degree and above	1,143	0.91 (0.40, 2.07)	1.71 (0.87, 3.36)	2.03 (0.94, 4.36)	2.51 (1.24, 5.07)	0.018
Marital status						
Single/separated/widowed/divorced	2,946	1.28 (0.84, 1.97)	1.50 (0.87, 2.60)	1.62 (0.81, 3.22)	2.16 (1.06, 4.38)	0.153
Married	2,451	1.39 (0.77, 2.51)	1.55 (0.95, 2.53)	1.68 (0.85, 3.33)	2.99 (1.49, 6.00)	0.044
Parity						
First infant	865	1.08 (0.63, 1.84)	1.45 (0.86, 2.44)	1.23 (0.62, 2.44)	1.46 (0.71, 3.01)	0.657
2 or more	4,532	1.54 (1.11, 2.14)	1.96 (1.32, 2.92)	2.43 (1.41, 4.21)	4.29 (2.59, 7.09)	<0.001
Preterm birth						
Yes (less than 37 weeks)	705	1.36 (0.97, 1.90)	1.77 (1.13, 2.79)	1.98 (1.09, 3.60)	3.34 (1.96, 5.69)	<0.001
No (37 weeks and above)	4,617	1.65 (1.06, 2.59)	2.15 (1.32, 3.48)	2.61 (1.37, 4.96)	3.75 (2.06, 6.83)	<0.001
Low birth weight						
Yes (less than 2500 grams)	456	1.32 (0.58, 2.98)	1.50 (0.44, 5.15)	2.25 (0.68, 7.44)	4.62 (1.70, 12.54)	0.036
No (2500 grams and above)	4,941	1.46 (1.07, 2.00)	1.94 (1.39, 2.72)	2.13 (1.34, 3.38)	3.40 (2.11, 5.49)	<0.001
Insurance						
Private or MediGap	2,112	1.62 (0.94, 2.81)	1.57 (0.89, 2.77)	1.80 (1.03, 3.16)	3.62 (1.94, 6.76)	0.001
Medicaid, CHIP, or a state-sponsored plan	1,600	1.16 (0.71, 1.90)	2.43 (1.09, 5.45)	1.21 (0.44, 3.36)	1.70 (0.40, 7.25)	0.278
Medicare, military, or other gv’t plans	328	1.95 (0.77, 4.95)	1.90 (0.82, 4.37)	2.87 (1.09, 7.54)	0.56 (0.93, 3.41)	0.004
Single service plan, IHS, or no coverage	1,357	1.08 (0.62, 1.88)	2.19 (0.99, 4.85)	3.77 (1.55, 9.16)	1.64 (0.72, 3.71)	0.004
Dwelling						
Urban, principal city	2,275	1.22 (0.72, 2.07)	3.21 (1.98, 5.22)	2.80 (1.34, 5.87)	5.68 (2.48, 12.97)	<0.001
Urban, other than principal city	2,365	1.95 (1.31, 2.90)	1.54 (0.94, 2.52)	1.87 (1.01, 3.48)	3.11 (1.70, 5.70)	<0.001
Rural	757	1.04 (0.49, 2.20)	1.51 (0.66, 3.41)	2.63 (1.06, 6.50)	2.61 (0.94, 7.27)	0.255

Note: based on 5,397 mothers with infants from the National Survey of Family Growth 2011–2013. Preterm data were missing for 75 participants and the number and percentage of breastfeeding by these participants are not presented in the table.

The separate logistic regressions tested the relationship between breastfeeding and Federal Poverty Level categories of household income for each characteristic.

CHIP: Children’s Health Insurance Program; gv’t: government; IHS: Indian Health Service; Wt Col%: weighted column percentage. FPL = Federal Poverty Level.

0–99% Federal Poverty Level is the reference group.

**Table 3:** Adjusted odds ratios (AOR) and 95% confidence intervals (CI) from logistic regression on breastfeeding National Survey of Family Growth (NSFG) 2011–2013 (*n* = 5397).

Adjusted logistic regression	Model 1	*p* value	Model 2	*p* value
Federal Poverty Level				
0–99% of Federal Poverty Level	Reference		Reference	
100–199% of Federal Poverty Level	1.23 (0.99, 1.70)	0.2091	1.19 (0.86, 1.65)	0.305
200–299% of Federal Poverty Level	1.24 (0.86, 1.79)	0.2392	1.25 (0.88, 1.76)	0.212
300–399% of Federal Poverty Level	1.09 (0.68, 1.75)	0.7081	1.05 (0.65, 1.70)	0.831
400% and above	1.50 (0.89, 2.51)	0.1281	1.45 (0.83, 2.52)	0.193
Maternal age				
14 to less than 20 years	0.76 (0.57, 1.00)	0.0467	0.75 (0.56, 1.00)	0.050
20 to less than 25 years	Reference		Reference	
25 to less than 30 years	1.18 (0.96, 1.44)	0.1180	1.17 (0.95, 1.43)	0.137
30 to 44 years	1.56 (1.15, 2.13)	0.0008	1.61 (1.18, 2.18)	0.002
Maternal race/ethnicity				
Non-Hispanic white			Reference	
Non-Hispanic black			0.52 (0.38, 0.71)	0.004
Hispanic			1.47 (1.03, 2.08)	0.516
Maternal education				
Less than high school	Reference		Reference	
High school graduate	0.53 (0.35, 0.79)	0.0019	0.61 (0.39, 0.98)	0.039
Some higher education	0.88 (0.59, 1.33)	0.5527	1.10 (0.69, 1.76	0.693
Associate degree and above	1.50 (0.99, 2.27)	0.0569	1.87 (1.16, 3.00)	0.010
Marital status				
Single/separated/widowed/divorced	0.65 (0.50, 0.84)		0.89 (0.73, 1.083)	0.221
Married	Reference		Reference	
Parity				
First infant			Reference	
2 or more			0.91 (0.71, 1.16)	0.432
Low birth weight				
Yes (less than 2500 grams)			0.86 (0.55, 1.33)	0.486
No (2500 grams and above)			Reference	
Preterm birth				
Yes(less than 37 weeks)			Reference	
No (37 weeks and above)			1.21 (0.93, 1.57)	0.164
Insurance				
Private or MediGap			0.85 (0.60, 1.22)	0.383
Medicaid, CHIP, or a state-sponsored plan			0.61 (0.44, 0.85)	0.003
Medicare, military, or other government plans			1.02 (0.68, 1.55)	0.909
Single service plan, Indian Health Service, or no coverage			Reference	
Dwelling				
Urban, principal city	1.56 (1.06, 2.29)	0.0243	1.62 (1.07, 2.46)	0.023
Urban, other than principal city	1.55 (1.11, 2.17)	0.0103	1.48 (1.04, 2.09)	0.028
Rural	Reference		Reference	

Note: based on 5,397 participants with infants from the National Survey of Family Growth 2011–2013.

**Table 4:** Adjusted stratified logistic regression results with race/ethnicity, gestation, and marital status on breastfeeding, National Survey of Family Growth (NSFG) 2011–2013.

		Adjusted odds ratios (95% confidence interval)				
	Number	100–199% FPL	200–299% FPL	300–399% FPL	400%+ FPL	*p* value
Maternal race/ethnicity						
Non-Hispanic white^1^	2,337	1.01 (0.61, 1.70)	0.93 (0.53, 1.61)	0.83 (0.44, 1.59)	1.00 (0.52, 1.92)	0.974
Non-Hispanic black^2^	1,313	1.23 (0.80, 1.90)	1.67 (0.90, 3.11)	1.96 (0.79, 4.88)	1.81 (0.84, 3.91)	0.253
Hispanic^3^	1,747	1.17 (0.62, 2.19)	1.21 (0.47, 3.09)	0.83 (0.39, 1.76)	2.49 (0.92, 6.76)	0.219
Marital status						
Single/separated/widowed/divorced^4^	2,946	1.15 (0.79, 1.68)	1.28 (0.71, 2.32)	1.18 (0.54, 2.56)	1.28 (0.58, 2.84)	0.892
Married^5^	2,451	1.16 (0.64, 2.09)	0.94 (0.53, 1.66)	0.90 (0.43, 1.87)	1.18 (0.58, 2.37)	0.869
Preterm birth						
Yes (less than 37 weeks)^6^	705	1.14 (0.64, 2.04)	1.14 (0.66, 1.98)	1.13 (0.51, 2.53)	1.44 (0.66, 3.13)	0.918
No (37 weeks and above)^7^	4,617	1.15 (0.85, 1.58)	1.26 (0.82, 1.93)	0.98 (0.56, 1.73)	1.40 (0.72, 2.75)	0.759
Interactions						
Non-Hispanic white, married, not preterm	544	0.60 (0.20, 1.75)	0.64 (0.17, 2.34)	0.64 (0.16, 2.61)	0.81 (0.19, 3.46)	0.864
Non-Hispanic white, single, not preterm	311	0.86 (0.39, 1.90)	0.84 (0.31, 2.32)	0.32 (0.08, 1.27)	0.43 (0.09, 2.08)	0.441
Non-Hispanic white, married, preterm	857	1.12 (0.39, 3.22)	0.56 (0.20, 1.59)	0.71 (0.22, 2.32)	0.86 (0.25, 2.95)	0.866
Non-Hispanic white, single, preterm	625	1.08 (0.57, 2.04)	3.00 (1.39, 6.48)	1.61 (0.54, 4.83)	1.08 (0.37, 3.12)	0.086
Non-Hispanic black, married, not preterm	89	0.76 (0.02, 27.42)	1.51 (0.01, 141.84)	1.89 (0.04, 91.30)	1.03 (0.01, 113.05)	0.987
Non-Hispanic black, single, not preterm	334	1.37 (0.45, 4.12)	0.76 (0.20, 2.86)	7.02 (0.79, 62.55)	0.72 (0.13, 4.02)	0.232
Non-Hispanic black, married, preterm	208	2.09 (0.55, 7.93)	2.43 (0.49, 12.01)	14.20 (1.61, 125.07)	6.08 (0.66, 55.59)	0.083
Non-Hispanic black, single, preterm	682	1.42 (0.76, 2.66)	2.28 (0.79, 6.59)	1.38 (0.38, 4.99)	2.04 (0.50, 8.36)	0.547
Hispanic, married, not preterm	204	*Limited cell sizes*	*Limited cell sizes*	*Limited cell sizes*	*Limited cell sizes*	
Hispanic, single, not preterm	250	0.83 (0.33, 2.08)	0.07 (0.01, 0.36)	*Limited cell sizes*	*Limited cell sizes*	
Hispanic, married, preterm	549	0.82 (0.50, 1.32)	1.60 (0.48, 5.35)	0.29 (0.07, 1.29)	1.11 (0.27, 4.58)	0.327
Hispanic, single, preterm	744	1.04 (0.43, 2.50)	2.02 (0.59, 6.92)	1.15 (0.28, 4.74)	1.42 (0.27, 7.42)	0.834

FPL = Federal Poverty Level.

0–99% Federal Poverty Level is the reference group.

Subgroups are adjusted for maternal age (14 to less than 20 years; 20 to less than 25 years; 25 to less than 30 years; or 30 to 44 years), maternal race/ethnicity (non-Hispanic white; non-Hispanic black; or Hispanic), maternal education (less than high school; high school graduate; some higher education; or Associate degree and above), marital status (married or single/separated/divorced), parity (first infant or 2 or more), preterm birth (yes, less than 37 weeks, or no, 37 weeks and above), low birth weight (yes, less than 2500 grams, or no, 2500 grams and above), insurance (private or MediGap; Medicaid, CHIP, or a state-sponsored plan; Medicare, military, or other government plans; or single service plan, IHS, or no coverage), and dwelling (urban, principal city; urban, other than principal city; or rural) except as noted.

1 Stratified to non-Hispanic white participants.

2 Stratified to non-Hispanic black participants.

3 Stratified to Hispanic participants.

4 Stratified to single/separated/widowed/divorced participants.

5 Stratified to married participants.

6 Stratified to participants with preterm infants.

7 Stratified to participants with infants who were not preterm.

## References

[1] Eidelman AI, Schanler RJ (2012). Policy statement. Breastfeeding and the use of human milk. Pediatrics.

[2] Healthy People 2020 (2015). Maternal, Infant, and Child Health 2020 Topics & Objectives. http://www.healthypeople.gov/2020/topics-objectives/topic/maternal-infant-and-child-health/objectives.

[3] Dinour LM, Pope GA, Bai YK (2015). Breast milk pumping beliefs, supports, and barriers on a university campus. Journal of Human Lactation.

[4] Dixit A, Feldman-Winter L, Szucs KA (2015). ‘Frustrated,’ “depressed,” and “devastated” pediatric trainees: US academic medical centers fail to provide adequate workplace breastfeeding support. Journal of Human Lactation.

[5] Flaherman VJ, Hicks KG, Huynh J, Cabana MD, Lee KA (2014). Positive and negative experiences of breast pumping during the first 6 months. Maternal and Child Nutrition.

[6] Besore CT (2014). Barriers to breastfeeding for hispanic mothers. Breastfeeding Medicine.

[7] Ma X, Liu J, Smith M (2014). WIC participation and breastfeeding in south carolina: updates from PRAMS 2009–2010. Maternal and Child Health Journal.

[8] Hedberg IC (2013). Barriers to breastfeeding in the WIC population. The American Journal of Maternal Child Nursing.

[9] Gross TT, Powell R, Anderson AK, Hall J, Davis M, Hilyard K (2015). WIC peer counselors’ perceptions of breastfeeding in African American women with lower incomes. Journal of Human Lactation.

[10] Jensen E (2012). Participation in the supplemental nutrition program for women, infants and children (WIC) and breastfeeding: national, regional, and state level analyses. Maternal and Child Health Journal.

[11] Heck KE, Braveman P, Cubbin C, Chávez GF, Kiely JL (2006). Socioeconomic status and breastfeeding initiation among california mothers. Public Health Reports.

[12] Lutter CK, Morrow AL (2013). Protection, promotion, and support and global trends in breastfeeding. Advances in Nutrition.

[13] McDowell MM, Wang CY, Kennedy-Stephenson J (2008). Breastfeeding in the United States: Findings from the National Health and Nutrition Examination Surveys, 1999–2006.

[14] McDonald SD, Pullenayegum E, Chapman B (2012). Prevalence and predictors of exclusive breastfeeding at hospital discharge. Obstetrics & Gynecology.

[15] Nickel NC, Martens PJ, Chateau D (2014). Have we left some behind? Trends in socio-economic inequalities in breastfeeding initiation: a population-based epidemiological surveillance study. Canadian Journal of Public Health.

[16] CDC (2015). National Survey of Family Growth. 2011–2013 NSFG: Public Use Data Files, Codebooks, and Documentation.

